# Effects of women with gestational diabetes mellitus related weight gain on pregnancy outcomes and its experiences in weight management programs: a mixed-methods systematic review

**DOI:** 10.3389/fendo.2023.1247604

**Published:** 2023-11-20

**Authors:** Jing He, Kaili Hu, Binghua Wang, Hui Wang

**Affiliations:** ^1^Nursing Department, Tongji Hospital of Tongji Medical College of Huazhong University of Science and Technology, Wuhan, China; ^2^School of Nursing, Tongji Medical College of Huazhong University of Science and Technology, Wuhan, China

**Keywords:** gestational diabetes mellitus, gestational weight gain, weight management, mixed-methods, review

## Abstract

**Introduction:**

Proper controlling gestational diabetes mellitus (GDM)-related gestational weight gain (GWG) during pregnancy can optimize pregnancy outcomes and improve postpartum glucose homeostasis. This study aimed to explore the existing intervention programs, the effects on pregnancy outcomes, and the experiences of weight management for GDM-related GWG in women with GDM.

**Methods:**

This mixed-methods systematic review was retrieved from nine databases. The retrieval time was from the database construction to September 20, 2023, and all studies were published in English and Chinese. The included records used quantitative, qualitative, or mixed methods and reported original studies of weight-related intervention regimens, effects on pregnancy outcomes, and women’s experiences and perceptions. This review used a convergent segregated approach to synthesize and integrate research findings from Joanna Briggs Institute (JBI) mixed-methods systematic reviews.

**Results:**

There were 16 articles that met the inclusion criteria, and the articles came from seven different countries and included 23,997 women with GDM. The meta-analysis pooled outcomes for the incidence of weight gain exceeding the Institute of Medicine (IOM) recommendations after GDM diagnosis to delivery was 0.31% (95% CI 0.21-0.42). The effectiveness of GDM-related weight interventions in reducing weight gain after GDM diagnosis was supported by quantitative evidence. The GDM-related GWG below the IOM recommendations is a protective factor (OR=0.68, 95%CI 0.48-0.97) for large for gestational Age (LGA), and above the IOM recommendations is a risk factor (OR=1.62, 95%CI 1.15-2.27) for LGA. In addition, no significant statistical significance was found in the pooled outcomes of small for gestational age (SGA). Avoiding excessive weight gain helps to optimize neonatal birth weight, pregnancy outcomes, and maternal blood glucose levels. According to qualitative survey results, some women with GDM experienced weight stigma, and a positive relationship between healthcare providers and GDM women helped in weight management.

**Conclusion:**

Following a diagnosis of GDM, weight management interventions positively affected GWG and pregnancy outcomes. In order to improve compliance and safety of weight management in women with GDM, criteria and interventions for weight gain associated with GDM need to be further explored and improved.

**Systematic Review Registration:**

https://www.crd.york.ac.uk/PROSPERO/display_record.php?RecordID=404492, identifier CRD42023404492.

## 1 Introduction

Pregnancy causes weight gain and fat accumulation, and body weight appears to be important in islet cell function and glycemic control (1). This increased adiposity and fat deposition during pregnancy may reduce the physiological ability to compensate for insulin resistance and further exacerbate maternal hyperglycemia (2). Excessive gestational weight gain before the diagnosis of GDM is an important modifiable risk factor for GDM (3, 4). Furthermore, insulin sensitivity is reduced during pregnancy, exacerbating hyperglycemia, especially in the third trimester.

Gestational diabetes mellitus (GDM) is defined as any degree of glucose intolerance first detected during pregnancy (5). The recorded prevalence of GDM varies worldwide, ranging from 1–30% due to different diagnostic criteria and regional population characteristics, one of the most common complications in obstetrics (6). A hyperglycemic uterine environment has adverse impacts at all stages of fetal development. In the short term, GDM increases the risk of macrosomia, potentially leading to long-term endocrine status changes and an increased risk of metabolic diseases such as obesity and cardiometabolic disorders in women and offspring (7).

There is a strong link between gestational weight gain in women with GDM and several clinically important outcomes, including higher insulin requirements, cesarean delivery rates, and significantly higher rates of large for gestational age (LGA) (8, 9). According to a systematic review, 37% (95% CI 33–41%) of women with GDM gained excess weight (10). Gestational weight is crucial in the etiology of GDM, and excessive weight gain is common in patients with GDM, exacerbating adverse pregnancy outcomes. However, further research is required to determine whether limiting pregnancy weight gain and the appropriate range of weight gain benefits women with GDM.

Current studies mainly focus on interventions related to blood glucose management in women with GDM and rarely include weight management in the context of GDM (11). Meek et al. found that controlling pregnancy weight gain after diagnosis of GDM should be prioritized to optimize pregnancy outcomes and improve postpartum glucose homeostasis (12). It is not too late to provide lifestyle advice or interventions to improve weight management and pregnancy outcomes after the diagnosis of GDM. Furthermore, qualitative studies have reported that pregnant women with GDM with excessive weight gain are normal and lack attention and management of weight gain (13).

Promoting the management and intervention of GDM-related gestational weight gain (GWG) is complicated, as numerous factors are associated with weight gains, such as blood glucose levels, dietary habits, physical activity, and patient perceptions (14–16). We recommend that weight gain associated with GDM be included in the disease management strategy for GDM. However, more research is required to determine the effectiveness of nursing interventions to promote weight management in women with GDM. Therefore, this study aimed to conduct a mixed-methods systematic review of existing interventions, the effects on pregnancy outcomes, and the experiences and perceptions of weight management for GDM-related GWG in women with GDM.

## 2 Methods

We conducted a mixed-methods systematic review to extract qualitative GDM-related weight interventions, implementation modalities, and women’s weight management experiences and perceptions and quantitatively assess the impact of weight gain on pregnancy outcomes. We followed the recommendations of the Joanna Briggs Institute (JBI) Methodology for Mixed-Method Systematic Reviews (17). This review used the Preferred Items for Reporting of Systematic Reviews and Meta-Analyses (PRISMA) statement for preparing and reporting (18). This review has been registered with PROSPERO (CRD42023404492).

### 2.1 Search strategy and study selection

A limited preliminary search was conducted using PubMed to find studies on topics related to weight management in women following the diagnosis of GDM through delivery. Then a complete search strategy was developed by analyzing the MeSH terms in the title and abstract and the index terms used to describe the article. We searched nine databases, including PubMed, Embase (via Ovid), Cochrane Library, Web of Science, CINAHL (via EBSCO), PsycINFO (via EBSCO), WANFANG DATA, China National Knowledge Infrastructure (CNKI), and VIP Database for Chinese Technical Periodicals. The search strategy combines different concepts, including “gestational diabetes mellitus” and “weight gain”, “weight management”, “weight awareness”, and other concepts for free term and subject term database search (**Appendix I**). The retrieval time was from the database construction to September 20, 2023, and they were published in English and Chinese.

All searched studies were imported into EndNote v.X9 (Clarivate Analytics, PA, USA) for management, and duplicate content was removed. The titles and abstracts were screened independently by two reviewers based on the inclusion criteria. The titles and abstracts that met the criteria were examined in full text to determine the relevant studies. Any disagreements between the two reviewers during the selection process shall be resolved by consulting with the third reviewer. Peer-reviewed studies or unpublished dissertations with quantitative data (intervention studies, observational studies), qualitative data, and studies with mixed methodology research designs were eligible for inclusion. Research proposals, meetings, reviews, and case reports were all excluded.

### 2.2 Assessment of methodological quality

Two independent reviewers (JH and KH) critically assessed the quality of the jurisprudence in studies that were eligible for inclusion. The quality of included studies was assessed and reported using the JBI Critical Appraisal Checklist for RCT (19), Cohort Studies (20), and Qualitative Research (21). The quantitative and qualitative components of mixed method studies were evaluated separately using appropriate JBI critical appraisal instruments. All items on the list were listed as yes, no, unclear, or not applicable. The quality rating results were used to assess confidence in the evidence rather than to exclude studies. Any disagreements between the two reviewers (JH and KH) were resolved by discussing or consulting a third reviewer (HW). According to the critical appraisal quality evaluation results, the results of answering ‘yes’ in 6 articles (37.5%) were 90% or above (**Appendix II**).

### 2.3 Data extraction

The extracted data included study-specific information (title, year of publication, authors, country), diagnostic information of participants, research methodology, and key results and findings related to the review questions. The draft data extraction tool was piloted in two or three studies. The extraction tool was modified and revised on a pilot basis before extracting data from the included evidence sources. Researchers constantly extracted data and updated them to prepare in chart forms and ensured that the extraction methods were consistent with the purpose of the study. Two independent reviewers used a developed data extraction tool to extract data from papers included in the scope of the review. Any differences between reviewers were resolved through discussion or negotiation with other reviewers. If required, the authors of those papers were contacted to request missing or additional data.

### 2.4 Data synthesis and integration

This review followed a convergent segregated approach, synthesizing and integrating data using the JBI mixed methods systematic review methodology (17). First, among the included quantitative studies, studies with outcomes more than or equivalent to three were included for meta-analysis. Combined incidence (%) and 95% confidence interval (CI) were combined to assess the incidence of GDM-related GWG exceeding the Institute of Medicine (IOM) recommendations. Moreover, the odds ratio (OR) and 95% CI were combined to evaluate the relationship between GDM-associated GWG and LGA and small for gestational age (SGA). Random effects model analysis was used to combine the pooled effect size and forest plots were used to show all pooled outcomes (22). *I^2^* statistical analysis was used to evaluate the degree of heterogeneity, and the Chi-square test (*p <*0.01) was used to evaluate the presence of heterogeneity between studies (23). Due to the small number of included studies, no subgroup analysis was performed in this study. There are not sufficient other findings to conduct a narrative synthesis study in quantitative studies. For all statistical analyses, Stata v.17.0 (Stata Corp LLC, Texas) was utilized.

Furthermore, the limited amount of qualitative research makes it impossible for all the terms to use text pools. Therefore, narrative synthesis is also used to present qualitative research results. The results of mixed method studies are reported separately into quantitative and qualitative components. Finally, quantitative, qualitative, and mixed methods evidence was integrated through narrative synthesis.

### 2.5 Patient and public involvement

Patients and/or the public were not involved in the design, implementation, reporting, or dissemination plan of this study. We used publicly available research and data for our analysis.

## 3 Results

**Figure 1:**
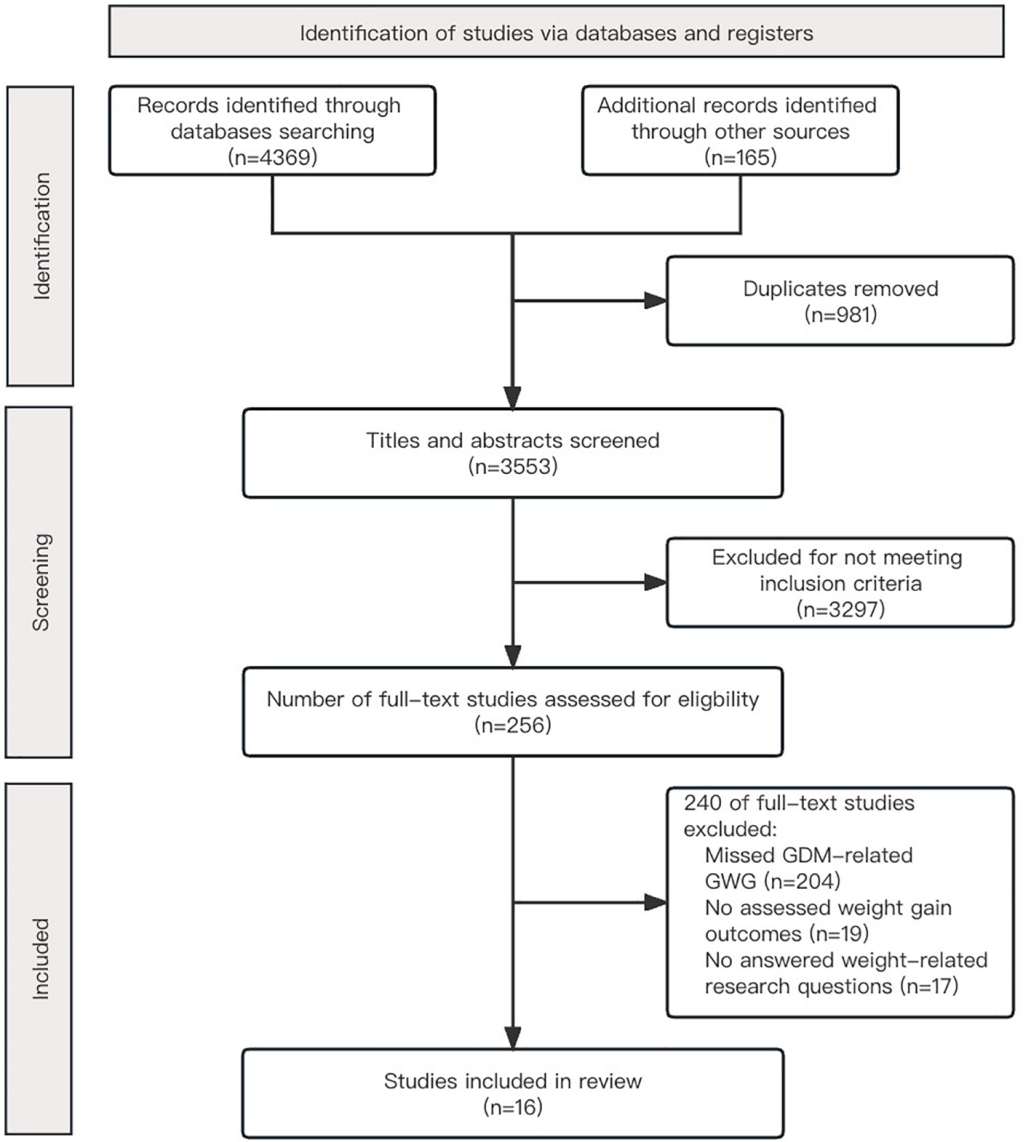
Flowchart of study selection.

We identified 16 articles in the present review that met the inclusion criteria following the search strategy generated and other sources of 4,534 studies (**Figure 1**). The included studies were published between 2015 and 2023. The number of participants ranged from 12 to 11,168, for a total of 23,997 pregnant women with GDM. Articles were primarily based in China (n=5), the United States (n=4), the UK (n=3), Australia, Saudi Arabia, Israel, and Singapore. There were 12 quantitative studies, nine of which were cohort studies and three were randomized controlled trials (RCT), three qualitative studies, and one mixed-methods study. **Table 1** depicts the characteristics and results of the included studies.

**Table 1:** Characteristics of included studies.

Author (year) and country	Treatment and blood glucose control	Study design		Study data collection time		Sample size	Diagnostic criteria for GDM			Research outcomes
Hong et al. (2022) (24) China	/	Retrospective cohort		January 2017 -January 2021		N=5275	IADPSG			LGA, macrosomia, SGA, and LBW
Barnes et al. (2022) (25) Australia	Diet and physical activity therapy or combined with insulin. Insulin use: below 103/283, Within, 186/449, Above personalized weight150/302.	Cohort		March 2016 -march 2019		N=1034	WHO			Therapeutic outcome (insulin use, total insulin dose units, basal bolus regimen, basal only insulin dose units), LGA, SGA, caesarean section, neonatal hypoglycaemia, early delivery, and ethnicity
Guo et al. (2019) (16) China	Diet and physical activity Hemoglobin A1C before delivery (%): 5.3 ± 0.3 vs 4.7 ± 0.2, P< 0.001. Percentage of off-target fasting glucose measurement (%), 8.3 ± 0.6 vs 4.6 ± 0.4mmol/L, P< 0.001. Percentage of off-target 2 h post-prandial glucose measurement (%), 14.7 ± 0.8 vs 7.9 ± 0.7, P< 0.001.	Randomized controlled trial (RCT)		December 2015 -December 2017		N=124	IADPSG			Compliance, frequency of outpatient service, The value of HbAlc at diagnosis, Hemoglobin A1C before delivery, percentage of off-target fasting glucose measurement, percentage of off-target 2 h post-prandial glucose measurement, OGTT-fasting value at diagnosis, OGTT-120 min value at diagnosis, OGTT—fasting value after 3 months of delivery, OGTT-120 min value after 3 months of delivery, normal vaginal delivery, instrumental delivery, episiotomy, shoulder dystocia, cesarean delivery, hypoglycemia of the newborn, fetal macrosomia, and gestational age at delivery
Chakkalakal et al. (2019) (26) USA	Glyburide and/ or insulin: 12/40, both treatments of which are associated with weight gain; sensitivity analysis excluding the 12 women, the results were similar	Prospective observational cohort		July 2014 - December 2015		N=40	ADA			GDM has a lower weight gain than non-GDM
Al-ofi et al. (2019) (14) Saudi Arabia	Diet and physical active Hemoglobin A1C before delivery (%): 5.3 ± 0.3 vs 4.7 ± 0.2, P< 0.001. Percentage of off-target fasting glucose measurement (%), 8.3 ± 0.6 vs 4.6 ± 0.4mmol/L, P< 0.001. Percentage of off-target 2 h post-prandial glucose measurement (%), 14.7 ± 0.8 vs 7.9 ± 0.7, P< 0.001.	RCT		Na		N=57	IADPSG			Fasting glucose, 2 h post-prandial glucose, and HbAlc
Aiken et al. (2019) (27) UK	Insulin only at 36 weeks, 40/144 Metformin and insulin at 36 weeks, 31/144	Retrospective observational		October 2014 - March 2017		N=376	IADPSG			LGA, SGA, vaginal delivery, caesarean delivery, instrumental delivery, dietary management only at 36 weeks, on metformin at 36 weeks, on insulin at 36 weeks, birthweight z score, post-partum fasting glucose, post-partum 2-h OGTT glucose, total daily insulin dose (36 weeks), total insulatard dose (36 weeks), and total novorapid dose (36 weeks)	
Komem et al. (2018) (2) Israel	Pharmacological treatment, Lost 43/124 Below 73/192 Within 16/45 If glucose threshold were met at more than 80% of measurement then glucose control was considered to be good glycemic control. Good glucose control, Lost96/124, Below132/192 Within29/45.	Retrospective cohort		July 2012 - December 2016		N=451	ACOG			Good glycemic control, pharmacological treatment, oligohydramnios, polyhydramnios, hypertensive disorders, mode of delivery, spontaneous vaginal delivery, assisted vaginal delivery, cesarean delivery, birthweight, grams, birthweight, percentile, LGA, SGA, neonatal jaundice, neonatal hypoglycemia, shoulder dystocia, and respiratory distress syndrome	
Hedderson et al. (2018) (28) USA	/	A cluster-randomized controlled trial		March 2011 -March 2012		N=2014	ACOG			Preterm birth, cesarean delivery, neonatal intensive care unit admission, SGA, and LGA	
Wang et al. (2015) (29) China	Diet and physical activity	Retrospective		June 20th - November 30th, 2013		N=2750	IADPSG			Preterm birth, macrosomia, LBW, and cesarean section	
Harper et al. (2015) (30) USA	Required Glyburide, Gain Less than IOM Recommendations55/175, within 22/92, more IOM 162/368; required insulin, gain less than IOM 3, with IOM 7, more IOM 14; Weight gain is associated with a higher risk of needing medication	Retrospective cohort		2007 - 2012		N=635	ACOG			Preeclampsia, all cesarean (primary and repeat), primary cesarean, A2 GDM, required glyburide, required insulin, time to blood sugar control, birth weight, macrosomia, LGA, SGA, preterm birth, and birth injury	
Surendran et al. (2021) (15) Singapore	Diet and physical activity therapy or combined with insulin	RCT and qualitative		June 2019 - January 2020		N=14	/			NA	
McParlin et al. (2019) (31) UK	/	Qualitative design		February - October 2015		N=12	/			NA	
Jarvie (2017) (32) UK	/	Qualitative		2011 - 2012		N=27	/			NA	
Nicholson et al. (2016) (33) USA	Glyburide or metformin Insulin 6/23, insulin 1/23), diet and physical activity	Qualitative		July, 2012 - April 2013		N=23	/			NA	
Lyu et al. (2023) (34) China	/	Retrospective cohort		2011 - 2021		N=11168	IADPSG			GDM-related gestational weight gain for underweight 0.51 (95% CI 0.44-0.58) kg/weeks, normal weight, 0.42 (0.35-0.50) kg/weeks, overweight 0.28 (0.23-0.33) kg/weeks, and obesity 0.22 (0.17-0.27) kg/weeks	
Liu et al. (2023) (35) China	/	Retrospective cohort		2011 - 2021		N=11168	IADPSG			LGA and macrosomia	

IADPSG, International Diabetes and Pregnancy Study Group; WHO, World Health Organization; ADA, American Diabetes Association; ACOG, The American College of Obstetricians and Gynecologists; GDM, gestational diabetes mellitus; LGA, large-for-gestational-age; SGA, small-for-gestational-age; LBW, low birth weight; RCT, randomized controlled trial; NA, not applicable; HbAlc, glycosylated hemoglobin; OGTT, oral glucose tolerance test.

Given the diversity of types in these researches, different intervention programs, and pregnancy outcomes, we present the results of the narrative review based on our four research objectives.

### 3.1 Nature of weight management interventions

Presently, the health information on weight management in women with GDM is primarily based on the education given by healthcare providers during antenatal care. However, it is difficult for patients to grasp this information. Mobile healthcare is a relatively new and emerging technology that has the potential to improve weight management awareness and reduce adverse effects on pregnancy outcomes in women with GDM. Four studies were included that provided routine care for gestational glucose and weight management combined with remote care for women with GDM (14–16, 28). There were differences in the content and delivery mode of the intervention. This study presented a narrative summary where all GDM-related gestational weight management and interventions were conducted in a mobile health format.

In order to analyze unusual results and provide feedback to healthcare providers, three (75%) studies developed an application that automatically uploads blood glucose results with the application linked to a glucose meter (14–16). The application also included health education courses and an interactive messaging platform. Guo et al. investigated intervention measures aimed primarily at blood glucose management and health education, including the influence of the remote intervention on GWG related to GDM and pregnancy outcome was observed (16). Al-ofi et al. also collected the weekly body weight of women with GDM by estimating the GWG target based on the chain in dynamic body weight to control body weight within the appropriate range (14). Surendran et al. also tracked physical activity, diet, and weight gain information (15).

Furthermore, Hedderson et al. (25%) used customized letters to guide weight management in women with GDM (28). They tailored an individual letter to each pregnant woman diagnosed with GDM based on electronic health record data. The letters included weight history, total GWG recommendations, weight goals, weight management advice based on weight trajectory, life tips, and statements about the risks of excessive gestational weight (28). Meanwhile, Hedderson et al. set new weight gain targets based on IOM recommendations due to the increased risk of complications in women with GDM (28). The pre-pregnancy body mass index (pre-BMI) of GDM women was found to be less than 18.5 kg/m^2^, not exceeding the midpoint of the IOM recommendation range of 18.5 kg/m^2^ or greater, and the recommendation for total GWG corresponds to the lower end of the IOM range (28). According to the above studies, M-health care can effectively control weight management gain, significantly improving adverse pregnancy outcomes in GDM women.

### 3.2 Management objectives and evaluation methods of GDM-related GWG

Three interventional studies reported weight management goals, and nine observational studies reported the methods GDM-related GWG was measured. In two intervention studies (2/3), weekly weight gain, weight gain trajectories, and weight management goals were tracked in women with GDM (14, 15). Hedderson et al. developed weight goals related to GDM based solely on pre-diagnosis weight gain and pre-pregnancy BMI (28). Guo et al. studied a remote intervention that did not involve weight but showed that the intervention group had lower mean weight gain associated with GDM than the control group (16). All three intervention studies showed effectiveness in weight management.

**Figure 2:**
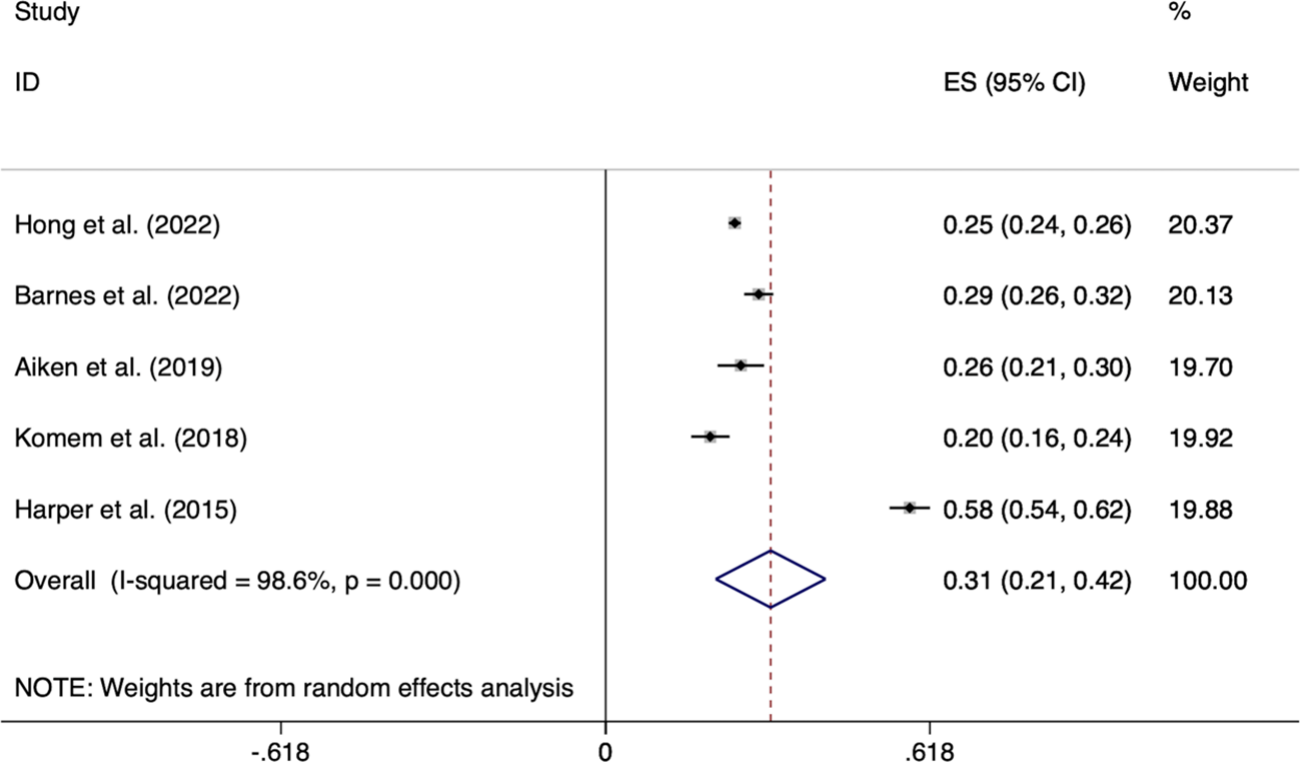
Forest plot of the incidence of GWG exceeding IOM recommendations after GDM diagnosis to delivery.

Furthermore, the included quantitative studies (10/11) used IOM criteria to guide GWG in women with GDM after diagnosis, but the measurement methods differed. Three studies (3/10) examined weight gain between the diagnosis of GDM and delivery (14, 25, 27). Five studies (6/10) evaluated GDM-related weight gain/corresponding gestational age equal to the weight growth rate. Changes in BMI associated with GDM were calculated in one study (2, 24, 26, 28, 30, 35). Wang et al. calculated BMI changes with GDM (29). According to the reports from five studies, the meta-analysis pooled outcomes for the incidence of weight gain exceeding IOM recommendations after GDM diagnosis to delivery was 0.31% (95% CI 0.21-0.42, *I^2^ =*98.6%, *p <*0.001), and shown in **Figure 2**.

Chakkalakal et al. compared the lower weight gain after GDM diagnosis to delivery than women without GDM, indicating that GDM treatment can improve weight management of pregnant mothers (26). In addition, a cohort study from China reported an optimal GDM-related GWG rate for women with GDM and different pre-BMI with the goal of reducing adverse birth weight outcomes (34). The recommended range of optimal GWG rates provided by this study is lower than the range recommended by the IOM (**Table 1**). Based on these findings, we learned that weight management after GDM diagnosis effectively controlled weight gain and reduced the incidence of excess weight gain and adverse outcomes compared to before diagnosis (24, 25, 30).

### 3.3 Effects of weight gain after GDM diagnosis on maternal-fetal outcome

#### 3.3.1 Neonatal weight outcomes of GDM-related GWG

**Figure 3:**
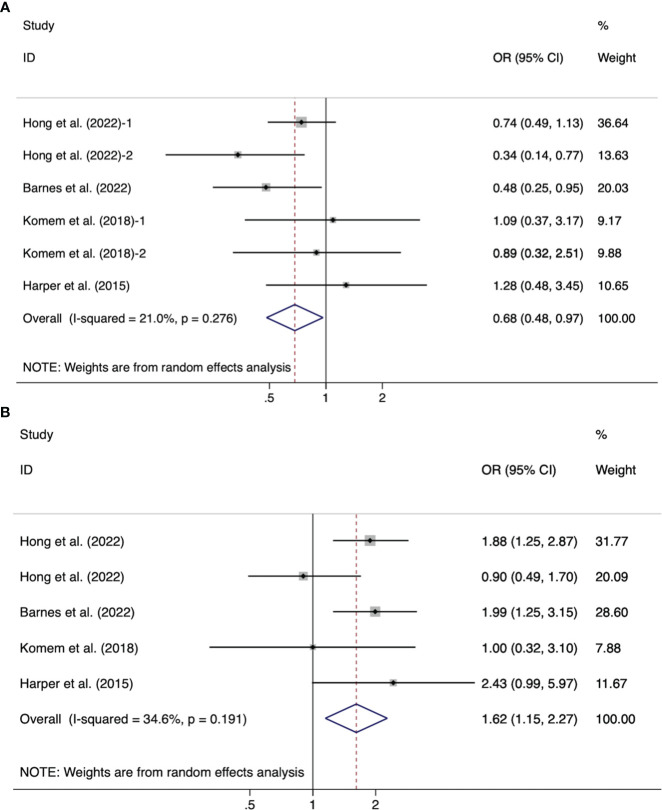
Forest plot of the association between the GDM-related GWG below **(A)**/above **(B)** the IOM recommendations for women with GDM and LGA.

**Figure 4:**
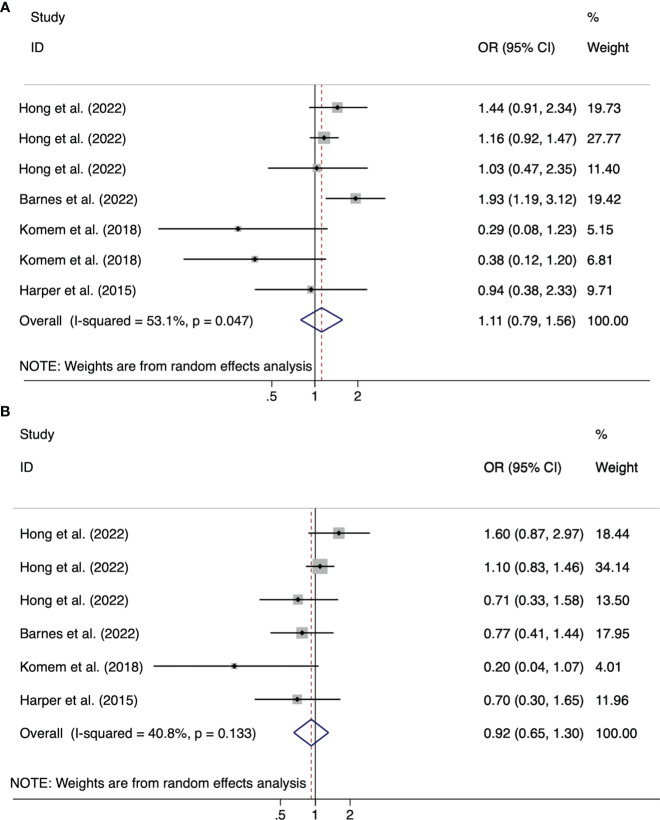
Forest plot of the association between the GDM-related GWG below **(A)**/above **(B)** the IOM recommendations for women with GDM and SGA.

Of the 16 articles, nine cohort studies and three intervention studies analyzed the impact of weight gain from GDM diagnosis to delivery (late pregnancy weight gain) on pregnancy outcomes. Effects of weight gain on neonatal birth weight included macrosomia, LGA, SGA, and LBW (**Table 2**). The pooled effect size of LGA for the GDM-related GWG below the IOM recommendations was OR=0.68 (95%CI 0.48-0.97, *I^2^ =*21%, *p* =0.28); the OR=1.62 (95%CI 1.15-2.27, *I^2^ =*34.6%, *p* =0.19) for LGA above the IOM recommendations (**Figure 3**). In addition, no significant statistical significance was found in the pooled outcomes of SGA, whether the GDM-related GWG was below (OR =1.11, 95%CI 0.79-1.56, *I^2^ =*53.1%, *p* =0.05) or above (OR =0.92, 95%CI 0.65-1.30, *I^2^ =*40.8%, *p* =0.13) the IOM recommendations (**Figure 4**).

**Table 2:** Effects of pregnant women with GDM-related GWG on neonatal birth weight.

Study	Characteristics of GWG analysis	Analytical method	LGA	Macrosomia	SGA	LBW
Hong et al. (2022) (24)	Weight growth rate compared with IOM: below, within, and above	Divided analysis of pre-pregnancy BMI, below and above (referent, within) (OR and 95% CI)	Normal weight: below 0.74 (0.49-1.13), above 1.88 (1.25-2.87); overweight/obesity: below 0.34 (0.14-0.77), above 0.90 (0.49-1.70)	Normal weight: below 0.54 (0.32-0.92), above 2.29 (1.43-3.72); overweight/obesity: below 0.31 (0.09-0.88), above 1.03 (0.49-2.28)	Underweight: below 1.44 (0.91-2.34), above 1.60 (0.87-2.97); normal weight: below 1.16 (0.92-1.47), above 1.10 (0.83-1.46); overweight/obesity: below 1.03 (0.47-2.35), above 0.71 (0.33-1.58)	Underweight: below 1.13 (0.44-3.32), above 1.64 (0.50-5.52); normal weight: below 1.20 (0.75-1.92), above 1.21 (0.75-1.94); overweight/obesity: below 4.77 (0.78-91.8), above 4.65 (0.85-86.7)
Barnes et al. (2022) (25)	Weight growth rate compared with IOM: below, within, and above	Below and above (referent, within) (OR and 95% CI)	Below 0.48 (0.25-0.95), above 1.99 (1.25-3.15)	/	Below 1.93 (1.19-3.12), above 0.77 (0.41-1.44)	/
Aiken et al. (2019) (27)	Weight gain form GDM diagnosis to delivery (kg)	Four classification method: a. continuous variable for weight gain; b. percentage gestational weight gain (referent 5-15%); c. minimum recommended gestational weight gain of 5 kg; d. gained <5kg (referent all other women) (OR and 95% CI)	a. 1.17 (1.01 to 1.37); b. <5% 0.30 (0.07 to 1.20), > 15% 2.83 (1.23 to 6.53); c. <5kg 0.43 (0.17 to 1.08), >5kg 1.57 (0.69 to 3.57); d. 0.18 (0.05 to 0.64)	/	a. 1.03 (0.88 to 1.20); b. <5% 2.43 (0.81 to 7.32), > 15% 0.55 (0.20 to 1.53); c. <5kg 6.85 (1.54 to 30.47), >5kg 4.14 (0.81 to 21.29); d. 2.27 (0.86 to 6.00)	/
Komem et al. (2018) (2)	Weight growth rate compared with IOM: below, within, and above	Four groups: lost, low, within, and above (case/all)	Lost 15/124, low 19/192, within 5/45, and above 10/90	/	Lost 4/124, low 8/192, within 5/45, and above 2/90	/
Harper et al. (2015) (30)	Weight growth rate compared with IOM: below, within, and above	Below and above (referent, within) (OR and 95% CI)	Below 1.28 (0.48-3.45), above 2.43 (0.99-5.97)	Below 0.46 (0.50-4.19), above 2.59 (0.98-6.84)	Below 0.94 (0.38-2.33), above 0.70 (0.30-1.65)	/
Guo et al. (2019) (16)	The weight difference between the two groups after intervention was compared	Control vs. Intervention	/	6/60 vs. 4/64	/	/
Hedderson et al. (2018) (28)	The weight difference between the two groups after intervention was compared	Control vs. Intervention	92/948 vs. 129/1008	/	82/943 vs. 79/1013	/
Liu et al. (2023) (35)	Weight gain form GDM diagnosis to delivery (kg/weeks)	Weekly GWG post-GDM diagnosis and the risk factors, (OR and 95% CI)	Underweight 3.02 (0.82-11.20), normal weight 2.64 (1.93-3.62), overweight 2.58 (1.73-3.82), obese 1.06 (1.03-1.08)	/	Underweight 6.48 (1.29-32.62), normal weight 3.30 (2.22-4.91), overweight 2.00 (1.22-3.26), obese 1.74 (0.75-4.04)	/

GDM, gestational diabetes mellitus; GWG, gestational weight gain; LGA, large-for-gestational-age; SGA, small-for-gestational-age; LBW, low birth weight; IOM, Institute of Medicine.

Furthermore, Hong et al. divided the weight gain of pregnant women into five intervals according to pre-BMI normal and overweight/obese women, observing both increased risk of macrosomia and LGA with weight gain using a dose-analysis method (24). Meanwhile, in females, normal weight, above the IOM guidelines, increased the risk of macrosomia 2.29 (1.43-3.72) (24). But in overweight/obese women, no risk was observed, and interestingly overweight/obese people have a higher incidence of exceeding the IOM guidelines than other women (27). And Barnes et al. found that women from the Middle East gained more weight and had a higher average infant birthweight than European women (25). Other studies have found that excessive weight gain does not affect macrosomia (2, 25, 29, 30) and LGA (2, 30).

Of the two telehealth management interventions, Guo et al. reported significantly lower weight gain in the intervention group than in the control group, but no effect on LGA was observed (16). Hedderson et al. observed a statistically significant weight growth only in those with a BMI ≥ 25 kg/m^2^, positively affecting LGA (9.7% vs. 12.8%, P = 0.04) (28). Aiken et al. reported that below GWG has a protective effect of macrosomia 0.54 (0.32-0.92) in women with normal weight, whereas below GWG has a protective effect of macrosomia 0.31 (0.09–0.88) for women with overweight/obesity (27). Furthermore, Aiken et al. found no difference in neonatal birthweight z score (27), whereas Komem et al. analyzed the lower birth weight of newborns who gained less weight than the recommended IOM (2).

#### 3.3.2 Pregnancy and neonatal outcomes of GDM-related GWG

The effects of GDM-related GWG on neonatal outcomes also included preterm birth (25, 28–30), gestational age (16), neonatal jaundice (2, 16), hypoglycemia (2, 16, 25), respiratory distress syndrome (2), birth injury (30), and NICU (28), but no statistically significant differences were observed for these outcomes. Among pregnancy outcomes, cesarean section (2, 16, 25, 27–30), vaginal delivery (2, 16, 27), instrumental delivery (16), oligohydramnios (2), and preeclampsia (30) were not associated with weight gain after GDM diagnosis. However, Aiken et al. analyzed the increased risk of instrumental delivery as GWG gradually increased (27). Moreover, women with GWG less than or equal to the recommended value had the lowest incidence of hypertension events and polyhydramnios compared to those within or above recommendations (2).

#### 3.3.3 Blood glucose levels and treatment outcome of GDM-related GWG

Conversely, GDM-related GWG may be associated with blood glucose levels and treatment outcomes. Total GWG influenced glucose control reasonably, but no correlation was found in GDM-related GWG (2). For two randomized controlled M-health intervention studies, GDM-related GWG in the intervention group was significantly lower than in the control group, and glycemic control was better with two-hour postprandial (14, 16). Gou et al. showed that the intervention group with low GWG had lower rates of pre-delivery glycated hemoglobin, percentage of off-target, and fasting glucose measurement (16). Furthermore, Al-ofi et al. found that better weight gain management in the intervention group was associated with blood glucose at two-hour postprandial before delivery but not with fasting blood glucose and Hba1c (14).

Barnes et al. reported a difference in the basal-bolus regimen exceeding the recommended GWG level compared to the within, but no risk relationship (25). The study also reported no statistical significance in the effects below, within, and above IOM recommendations of GDM-related GWG in insulin use, total insulin dose units, and basal-only insulin dose units (25). Aiken et al. analyzed no difference in weight gain between GDM diagnosis at 36 weeks and dietary management, metformin, and insulin. However, no differences were observed in total daily insulin, insulatard, and novorRapid doses (27). In this context, Harper et al. reported that weight gain beyond the IOM was associated with a higher risk of worse GDM (OR 2.55, 1.42–4.56) and the required glyburide treatment (OR 2.52, 1.39–4.56) than weight gain within the IOM (30).

### 3.4 Experiences and cognition of weight management

Two studies examined the feelings and experiences of pregnant women with GDM using a remote weight management system (15, 33). Pregnant women with GDM could share successful weight management strategies, get motivational health tips, learn about the relationship between weight and glucose metabolism, and learn how to control their weight (15, 28). Women with GDM also expressed that the weight tracking system’s simple and intuitive weight trajectory maps could improve their weight management awareness (33). In contrast, some women with GDM stated that weight management was not a priority and weight tracking caused anxiety (33).

Women with GDM who participated in the weight loss study reported a positive perception of weight loss during pregnancy because their baby’s weight remained within the normal range throughout the pregnancy in the weight-loss study (2, 31). Other women felt that weight management has helped them control and reduce blood glucose and fatty liver disease with a sense of accomplishment (31).

Conversely, Jarvie’s study understood the life experiences of obese (BMI > 30) women with GDM (32). Obese women generally believe that being told about their obesity status by healthcare professionals is shocking and shameful. Obesity is a stigmatized word, and “fat” sounds bad and hinders weight management.

Simultaneously, women feel stigmatized and unfairly blamed if healthcare professionals assume that their high BMI is due to being asked to take an oral glucose tolerance test (OGTT) test or tell them that GDM is directly related to their weight (15, 32). Healthcare professionals always attribute various problems with the birth examination to weight, commenting on weight and making obese pregnant women with GDM uncomfortable. Instead, some women with GDM discussed and developed good relationships with community midwives, who would praise them for making lifestyle changes and offer support when they felt stigmatized by their weight to promote weight management (32).

## 4 Discussion

The mixed-methods systematic review is the first to synthesize evidence of GWG in women with GDM from diagnosis to delivery using quantitative, qualitative, and mixed-methods by understanding the important effects of weight on pregnancy outcomes. The evidence includes interventions, the effects on pregnancy outcomes, and experiences and perceptions of GDM-related GWG. Few intervention programs related to GDM disease focus on gestational weight but more on blood glucose management. It is not a surprise that GDM occurs in a series of pregnancy outcomes caused by abnormal blood glucose levels. Therefore, studies on weight management interventions in women with GDM and their effects on pregnancy outcomes are still in their early stages.

The review identifies the mode, content, and effectiveness of weight interventions for GDM-related GWG. M-health/telehealth is a standard and effective implementation model for monitoring and managing blood glucose and weight in women with GDM (11). The applications could provide a record of disease-related information, such as blood glucose, dietary habits, body weight, general patient education, or scheduled reminders to reduce adverse events (14–16). Although each application addressed one or more GDM clinical pathways, only two included weight change tracking and weight goal setting to protect self-care (14, 15). Telemedicine studies on weight management, diet, and physical activity have shown that electronic behavioral interventions can promote a healthy lifestyle and prevent excessive weight gain during pregnancy for women with GDM (36, 37). Thus, there is a need for more research into the benefits of controlling weight gain for women with GDM and their offspring because only limited studies on guidelines and management strategies for GDM-related GWG are available.

Although the goals of weight management and the GDM-related GWG were reported in the intervention studies, the methods for setting and evaluating the goals were not presented consistently. While setting weight management goals, there are differences in whether the weight before the diagnosis of GDM is included in the later weight management, which may affect the outcome evaluation. Furthermore, the weight measurement included weight gain, weight gain rate, and BMI changes between the diagnosis of GDM and delivery, making it challenging to compare GDM-related GWG at different levels. However, studies must also consider the interaction of various complex factors on weight gain, such as GDM disease, pre-pregnancy weight, diet management, physical activity, and blood glucose levels (25, 38). Lyu et al. attempted to explore optimal GDM-related GWG based on Chinese populations, and found that giving GWG rates lower than IOM recommendations was beneficial to newborn birth weight (34). We must emphasize how crucial weight management is to the treatment of GDM disease and the well-being of women and children.

In this review, pre-pregnancy overweight/obese women exceeded IOM recommendations more than women in other BMI categories (24, 25). Moreover, GDM-related GWG exceeding the IOM recommendation was associated with higher neonatal birth weight and significantly increased risk of LGA and macrosomia in women with pre-pregnancy BMI normal and overweight/obese (24, 25, 27). In contrast, weight gain in women with normal pre-pregnancy BMI was lower than IOM guidelines and associated with a reduced risk of macrosomia (2, 25, 27). In overweight/obese women, much less than recommended levels were associated with a lower risk of LGA and macrosomia (39). These findings are similar to the meta-pooled results of our study, where excess GDM-associated GWG is associated with a high risk of LGA. Furthermore, better weight gain management was associated with the postprandial two-hours display of blood glucose before delivery (16). These findings support the effect of fat accumulation on β-cell function and insulin sensitivity, and further weight gain highlights the importance of glycemic control (1).

Simultaneously, women with GDM and GDM-related GWG may have lower GWG than recommended by the IOM guidelines, which may benefit maternal and infant health (40, 41). The pooled results of this study show that GWG below the IOM recommendations is a protective factor for LGA. And Hedderson et al. investigated intervention trials of individualized weight management goals, considering pre-pregnancy BMI and GWG at the time of diagnosis of GDM, and reported that no adverse pregnancy outcomes were found in women with GDM within the minimum recommended range of IOM (28). These findings further suggest that a lower GWG than IOM recommendations may be more beneficial for women with GDM. In order to promote the health of women with GDM and their offspring, it is critical to provide weight management programs, goals, and new knowledge.

Moreover, women with GDM have both positive and negative experiences and perceptions of weight management, which are important influencing factors of weight management. The main motivators for women with GDM to achieve self-weight management are infant health and well-being (13, 42). Recommendations for weight management/lifestyle changes that are unrealistic or difficult to implement can increase the adversely affected relationships of women with GDM and healthcare providers and exacerbate their weight stigma, especially for women with obesity (43, 44). Draffn et al. emphasized the importance of carefully considering the backgrounds and needs of women with GDM to avoid making them feel favored or alienated (45). Therefore, continuity of care and a close relationship with the patient can help with communication and weight management.

The review has some limitations. First, most of the included studies are based on observations, and the evaluation methods of GDM-related GWG vary. Further analysis was not available to determine the effect of GDM-related GWG on pregnancy outcomes. Second, the number of intervention studies is small, and the protocols differ, estimating the specific impact of the intervention on weight gain becomes impossible. This limitation highlights the need for further research and a better study design. Third, only studies published in English and Chinese were reviewed due to resource and language constraints. Reviewing studies, including other speaking samples, is necessary to assemble the required international evidence.

## 5 Conclusion

The present review reported that weight management interventions after diagnosis of GDM had a positive effect on GWG, increased knowledge of the impact of GDM-related GWG on pregnancy outcomes, and gained insight into the experiences and perceptions of weight management in women with GDM. GDM causes significant harm to the mother and infant, but this harm can be minimized by improving weight gain. However, there is still a lack of recommendations and standards for weight gain associated with GDM and the awareness of the risks of reducing weight gain. In the future, we should explore the appropriate range of weight gain related to GDM and weight management intervention programs and pay attention to the personal feelings of women with GDM for women with different pre-pregnancy BMI.

## Data availability statement

The original contributions presented in the study are included in the article/**Supplementary Material**. Further inquiries can be directed to the corresponding author.

## Author contributions

JH, KH, BW, and HW conceived the study. JH, and KH performed data extraction and quality assessment of the included studies. JH, and BW carried out the statistical analysis of the data. JH drafted the manuscript. HW provided research supervision and critically modified the content of the manuscript. All authors read and approved the final manuscript.
