# Supplementary material for: Effects of women with gestational diabetes mellitus related weight gain on pregnancy outcomes and its experiences in weight management programs: a mixed-methods systematic review
Source: Front Endocrinol (Lausanne). 2023 Nov 20;14:1247604. doi: 10.3389/fendo.2023.1247604 (PMC10699134; doi:10.3389/fendo.2023.1247604)
Supplement: Supplementary file 1 [file DataSheet_1.docx]

**Appendix I: Search strategy**

Search was conducted on September 20, 2023

**PubMed**

| Search | Query | Records retrieved |
| --- | --- | --- |
| #1 | “Diabetes, Gestational”[Title/Abstract] | 381 |
| #2 | “Diabetes, Pregnancy Induced”[Title/Abstract] | 44 |
| #3 | “Pregnancy Induced Diabetes”[Title/Abstract] | 19 |
| #4 | “Gestational Diabetes”[Title/Abstract] | 21,494 |
| #5 | “Diabetes Mellitus, Gestational”[Title/Abstract] | 85 |
| #6 | “Gestational Diabetes Mellitus”[Title/Abstract] | 12,977 |
| #7 | GDM[Title/Abstract] | 11,457 |
| #8 | “Pregnancy Diabetes Mellitus”[Title/Abstract] | 72 |
| #9 | “Diabetes in Pregnancy”[Title/Abstract] | 1518 |
| #10 | #1 OR #2 OR #3 OR #4 OR #5 OR #6 OR #7 OR #8 OR #9 | 23124 |
| #11 | Weight[Title/Abstract] | 984,363 |
| #12 | “Body Weight”[Title/Abstract] | 243,342 |
| #13 | “Body Weights”[Title/Abstract] | 12,245 |
| #14 | “Weight*, Body”[Title/Abstract] | 10,522 |
| #15 | “Weight Management”[Title/Abstract] | 8,798 |
| #16 | “Weight Control”[Title/Abstract] | 7,360 |
| #17 | “Weight Management Knowledge”[Title/Abstract] | 6 |
| #18 | “Body Weight Changes”[Title/Abstract] | 1,951 |
| #19 | “Body Weight Change”[Title/Abstract] | 1,481 |
| #20 | “Change*, Body Weight”[Title/Abstract] | 97 |
| #21 | “Weight Change* Body”[Title/Abstract] | 42 |
| #22 | “Weight Management Needs”[Title/Abstract] | 12 |
| #23 | “Weight Knowledge”[Title/Abstract] | 21 |
| #24 | "Weight Management Experience*"[Title/Abstract] | 17 |
| #25 | “Weight Attitude”[Title/Abstract] | 5 |
| #26 | “Weight Belief”[Title/Abstract] | 4 |
| #27 | #11 OR #12 OR #13 OR #14 OR #15 OR #16 OR #17 OR #18 OR #19 OR #20 OR #21 OR #22 OR #23 OR #24 OR #25 OR #26 | 962,114 |
| #28 | #10 AND #27 | 4533 |

**CINAHL**

S1 Su(“Diabetes, Gestational” Or “Diabetes, Pregnancy Induced” Or “Pregnancy Induced Diabetes” Or “Gestational Diabetes” Or “Diabetes Mellitus, Gestational” Or “Gestational Diabetes Mellitus” Or Gdm Or “Pregnancy Diabetes Mellitus” Or “Diabetes In Pregnancy”)

S2 Su(Weight Or “Body Weight” Or “Body Weights” Or “Weight*, Body” Or “Weight Management” Or “Weight Control” Or “Weight Management Knowledge” Or “Body Weight Changes” Or “Body Weight Change” Or “Change*, Body Weight” Or “Weight Change* Body” Or “Weight Management Needs” Or “Weight Knowledge” Or “Weight Needs” Or “Weight Management Experience*” Or “Weight Management Feel*” Or “Weight Attitude” Or “Weight Belief”)

S3 SU(S1 AND S2)

**Embase (via Ovid)**

#1 'diabetes, gestational'/exp OR 'diabetes, gestational' OR 'diabetes, pregnancy induced' OR 'pregnancy induced diabetes' OR 'gestational diabetes'/exp OR 'gestational diabetes' OR 'diabetes mellitus, gestational' OR 'gestational diabetes mellitus'/exp OR 'gestational diabetes mellitus' OR gdm OR 'diabetes in pregnancy' OR 'pregnancy diabetes mellitus'/exp OR 'pregnancy diabetes mellitus'

#2 weight OR 'body weight' OR 'body weights' OR 'weight*, body' OR 'weight management' OR 'weight control' OR 'weight management knowledge' OR 'body weight changes' OR 'body weight change' OR 'change*, body weight' OR 'weight change* body' OR 'weight management needs' OR 'weight knowledge' OR 'weight needs' OR 'weight management experience*' OR 'weight management feel*' OR 'weight attitude' OR 'weight belief'

'diabetes, gestational' OR 'diabetes, pregnancy induced' OR 'pregnancy induced diabetes' OR 'gestational diabetes'/exp OR 'gestational diabetes' OR 'diabetes mellitus, gestational' OR 'gestational diabetes mellitus'/exp OR 'gestational diabetes mellitus' OR gdm OR 'diabetes in pregnancy' OR 'pregnancy diabetes mellitus'

#3 #1 AND #2

**APA Psycinfo (Via EBSCO)**

S1 SU(“Diabetes, Gestational” OR “Diabetes, Pregnancy Induced” OR “Pregnancy Induced Diabetes” OR “Gestational Diabetes” OR “Diabetes Mellitus, Gestational” OR “Gestational Diabetes Mellitus” OR GDM OR “Pregnancy Diabetes Mellitus” OR “Diabetes In Pregnancy”)

S2 SU(Weight OR “Body Weight” OR “Body Weights” OR “Weight*, Body” OR “Weight Management” OR “Weight Control” OR “Weight Management Knowledge” OR “Body Weight Changes” OR “Body Weight Change” OR “Change*, Body Weight” OR “Weight Change* Body” OR “Weight Management Needs” OR “Weight Knowledge” OR “Weight Needs” OR “Weight Management Experience*” OR “Weight Management Feel*” OR “Weight Attitude” OR “Weight Belief”)

S3 SU(S1 AND S2)

**Cochrane Library**

“Diabetes, Gestational” OR “Diabetes, Pregnancy Induced” OR “Pregnancy Induced Diabetes” OR “Gestational Diabetes” OR “Diabetes Mellitus, Gestational” OR “Gestational Diabetes Mellitus” OR GDM OR “Pregnancy Diabetes Mellitus” OR “Diabetes in Pregnancy” in Title Abstract Keyword AND Weight OR “Body Weight” OR “Body Weights” OR “Weight*, Body” OR “weight management” OR “weight control” OR “weight management knowledge” OR “Body Weight Changes” OR “Body Weight Change” OR “Change*, Body Weight” OR “Weight Change* Body” OR “Weight management needs” OR “weight knowledge” OR “Weight needs” OR “Weight management experience*” OR “Weight management feel*” OR “Weight attitude” OR “Weight belief” in Title Abstract Keyword - (Word variations have been searched)

**Web of Science**

((TS=(“Diabetes, Gestational” OR “Diabetes, Pregnancy Induced” OR “Pregnancy Induced Diabetes” OR “Gestational Diabetes” OR “Diabetes Mellitus, Gestational” OR “Gestational Diabetes Mellitus” OR GDM OR “Pregnancy Diabetes Mellitus” OR “Diabetes in Pregnancy”)) AND TS=(Weight OR “Body Weight” OR “Body Weights” OR “Weight*, Body” OR “weight management” OR “weight control” OR “weight management knowledge” OR “Body Weight Changes” OR “Body Weight Change” OR “Change*, Body Weight” OR “Weight Change* Body” OR “Weight management needs” OR “weight knowledge” OR “Weight needs” OR “Weight management experience*” OR “Weight management feel*” OR “Weight attitude” OR “Weight belief”))

**China National Knowledge Infrastructure (CNKI)**

SU=(“Diabetes, Gestational” OR “Diabetes, Pregnancy Induced” OR “Pregnancy Induced Diabetes” OR “Gestational Diabetes” OR “Diabetes Mellitus, Gestational” OR “Gestational Diabetes Mellitus” OR GDM OR “Pregnancy Diabetes Mellitus” OR “Diabetes in Pregnancy”) AND SU=(Weight OR “Body Weight” OR “Body Weights” OR “Weight*, Body” OR “weight management” OR “weight control” OR “weight management knowledge” OR “Body Weight Changes” OR “Body Weight Change” OR “Change*, Body Weight” OR “Weight Change* Body” OR “Weight management needs” OR “weight knowledge” OR “Weight needs” OR “Weight management experience*” OR “Weight management feel*” OR “Weight attitude” OR “Weight belief”)

**WANFANG DATA**

SU:(“Diabetes, Gestational” OR “Diabetes, Pregnancy Induced” OR “Pregnancy Induced Diabetes” OR “Gestational Diabetes” OR “Diabetes Mellitus, Gestational” OR “Gestational Diabetes Mellitus” OR GDM OR “Pregnancy Diabetes Mellitus” OR “Diabetes in Pregnancy”) AND SU:(Weight OR “Body Weight” OR “Body Weights” OR “Weight*, Body” OR “weight management” OR “weight control” OR “weight management knowledge” OR “Body Weight Changes” OR “Body Weight Change” OR “Change*, Body Weight” OR “Weight Change* Body” OR “Weight management needs” OR “weight knowledge” OR “Weight needs” OR “Weight management experience*” OR “Weight management feel*” OR “Weight attitude” OR “Weight belief”)

**VIP Database for Chinese Technical Periodicals**

M=(“Diabetes, Gestational” OR “Diabetes, Pregnancy Induced” OR “Pregnancy Induced Diabetes” OR “Gestational Diabetes” OR “Diabetes Mellitus, Gestational” OR “Gestational Diabetes Mellitus” OR GDM OR “Pregnancy Diabetes Mellitus” OR “Diabetes in Pregnancy”) AND M=(Weight OR “Body Weight” OR “Body Weights” OR “Weight*, Body” OR “weight management” OR “weight control” OR “weight management knowledge” OR “Body Weight Changes” OR “Body Weight Change” OR “Change*, Body Weight” OR “Weight Change* Body” OR “Weight management needs” OR “weight knowledge” OR “Weight needs” OR “Weight management experience*” OR “Weight management feel*” OR “Weight attitude” OR “Weight belief”)
