# Supplementary material for: Effects of women with gestational diabetes mellitus related weight gain on pregnancy outcomes and its experiences in weight management programs: a mixed-methods systematic review
Source: Front Endocrinol (Lausanne). 2023 Nov 20;14:1247604. doi: 10.3389/fendo.2023.1247604 (PMC10699134; doi:10.3389/fendo.2023.1247604)
Supplement: Supplementary file 2 [file DataSheet_2.docx]

Appendix II: Methodological quality of included studies (n=14)

| References | Q1 | Q2 | Q3 | Q4 | Q5 | Q6 | Q7 | Q8 | Q9 | Q10 | Q11 | Q12 | Q13 |
| --- | --- | --- | --- | --- | --- | --- | --- | --- | --- | --- | --- | --- | --- |
| Hong et al. ^a^  (2022) | Y | Y | Y | Y | Y | Y | Y | Y | Y | Y | Y | / | / |
| Barnes et al. ^a^  (2022) | Y | Y | Y | Y | Y | Y | Y | Y | Y | Y | Y | / | / |
| Guo et al. ^b^  (2019) | Y | Y | Y | Y | Y | Y | U | U | Y | Y | U | Y | Y |
| Chakkalakal et al. ^a^  (2019) | Y | Y | U | Y | Y | Y | Y | Y | Y | Y | Y | / | / |
| Al-ofi et al. ^b^  (2019) | Y | Y | Y | Y | Y | U | Y | Y | U | U | N | Y | Y |
| Aiken et al. ^a^  (2019) | Y | Y | U | Y | Y | Y | Y | Y | Y | Y | Y | / | / |
| Komem et al. ^a^  (2018) | Y | Y | U | Y | Y | Y | Y | Y | Y | Y | Y | / | / |
| Hedderson et al. ^b^  (2018) | Y | Y | Y | Y | Y | U | Y | Y | Y | Y | Y | Y | Y |
| Wang et al. ^a^  (2015) | Y | Y | N | N | N | Y | Y | Y | Y | Y | N | / | / |
| Harper et al. ^a^  (2015) | Y | Y | U | Y | Y | Y | U | Y | Y | Y | Y | / | / |
| Surendran et al. ^b, c^  (2021) | Y | Y | Y | Y | Y | Y | Y | Y | Y | Y | Y | / | / |
|  | U | U | Y | Y | Y | U | Y | Y | Y | Y | U | Y | N |
| McParlin et al. ^c^  (2019) | U | Y | Y | Y | Y | N | N | Y | Y | Y | / | / | / |
| Jarvie ^c^  (2017) | U | Y | Y | Y | Y | Y | U | Y | Y | Y | / | / | / |
| Nicholson et al. ^c^  (2016) | U | Y | Y | Y | Y | N | U | Y | Y | Y | / | / | / |
| Lyu et al. ^a^  (2023) | Y | Y | U | Y | Y | Y | Y | Y | Y | NA | Y | / | / |
| Liu et al. ^a^  (2023) | Y | Y | U | Y | Y | Y | Y | Y | Y | NA | Y | / | / |

a, cohort studies; b, RCTs; c, qualitative research; Y, yes; N, no; U, unclear, NA, not applicable

The quality of the included studies was assessed and reported using the Joanna Briggs Institute Critical Appraisal.
